# Consensus on the secondary prevention of primary liver cancer

**DOI:** 10.1007/s12072-021-10259-7

**Published:** 2021-11-30

**Authors:** Yuemin Nan, Xiaoyuan Xu, Yanhang Gao, Rongqi Wang, Wengang Li, Ming Yang, Lingdi Liu, Zhongping Duan, Jidong Jia, Lai Wei, Hui Zhuang, Chinese Society of Hepatology, Chinese Medical Association, Huiguo Ding, Zhongping Duan, Jiangao Fan, Qinmao Fang, Yanhang Gao, Peng Hu, Jidong Jia, Wengang Li, Jingfeng Liu, Junqi Niu, Yuemin Nan, Jia Shang, Rongqi Wang, Lai Wei, Yanyan Yu, Yuguo Zhang, Suxian Zhao, Jian Zhou, Weifeng Zhao, Xiaoyuan Xu, Chuanmiao Xie, Wen Xie, Ming Yang, Hui Zhuang

**Affiliations:** 1 Present Address: Department of Traditional and Western Medical Hepatology, The Third Hospital of Hebei Medical University, Shijiazhuang, 050051 China; 2 Department of Infectious Diseases, Peking University First Hospital, Beijing, 100034 China; 3 Department of Hepatology, The First Hospital of Jilin University, Changchun, 130021 China; 4 Radiation Oncology Centre, The Fifth Medical Centre of Chinese PLA General Hospital, Beijing, 100039 China; 5 Peking University Hepatology Institute, Peking University People’s Hospital, Beijing, China; 6 Artificial Liver Centre, Beijing You-An Hospital, Capital Medical University, Beijing, China; 7 Liver Research Centre, Beijing Friendship Hospital, Capital Medical University, Beijing, China; 8 Hepatopancreatobiliary Centre, Beijing Tsinghua Changgung Hospital, Tsinghua University, Beijing, China; 9 Department of Microbiology and Centre for Infectious Diseases, Peking University Health Science Centre, Beijing, China

**Keywords:** Liver neoplasms, Secondary prevention, Early detection of cancer

## Abstract

To standardize the effective prevention, surveillance, and diagnosis of primary liver cancer, the Chinese Society of Hepatology, Chinese Medical Association, invited clinical experts and methodologists to develop the *Consensus on the Secondary Prevention of Primary Liver Cancer*, which was based on the clinical and scientific advances on hepatocellular carcinoma. The purpose is to provide a current basis for the prevention, surveillance, and early diagnosis of primary liver cancer in patients with chronic liver diseases.

## Introduction

**Fig. 1:**
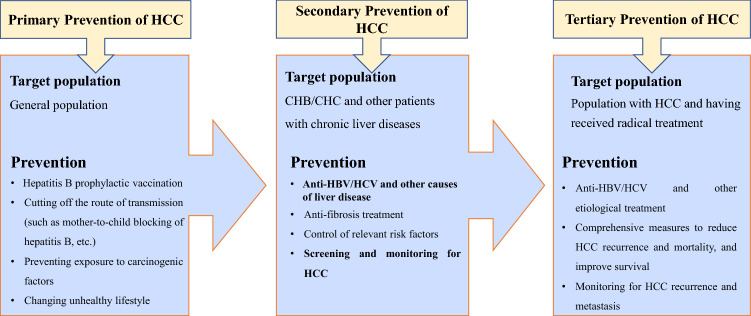
Target population and measures of the three levels of HCC prevention

The purpose of stratified prevention and surveillance of primary liver cancer is to identify and eliminate the risk factors that promote the progression of chronic liver disease. The following classification concept is adopted in the *Consensus*: primary prevention focuses on preventing initial harm to the general population from the risk factors; secondary prevention is to control the relevant causes and risk factors, and carry out risk-stratified surveillance in the population with chronic liver disease, thereby reducing or delaying the occurrence of HCC; tertiary prevention is to further reduce HCC recurrence and mortality, and improve overall survival after radical treatment (Fig. 1). With recent progress in basic and clinical research and the development of diagnostic techniques, the formulation of a consensus on secondary prevention of primary liver cancer will provide an important basis for prevention and control of liver cancer.

Primary liver cancer is a common malignant tumor and a significant cause of cancer deaths, and mainly includes three different pathological types: hepatocellular carcinoma (HCC), intrahepatic cholangiocarcinoma (ICC), and HCC–ICC, which are different in pathogenesis, biological behavior, histological morphology, treatment approaches, and prognosis. HCC accounts for 85–90% of primary liver cancers. Therefore, for the *Consensus*, “primary liver cancer” refers only to HCC.

## The consensus development process

The consensus is reached by a panel of experts consisting clinical epidemiologists, hepatologists, hepatobiliary surgeons, interventional radiologists, and oncologists, organized by the Chinese Society of Hepatology (CSH). The *consensus* is based on the current scientific evidence and practicing norms in the etiology, pathogenesis, diagnostic techniques, prevention, and treatment of primary liver cancer used in the clinical practice in the Asia–Pacific region and worldwide. The contents of the consensus have been refined by the panel through multiple rounds of discussions, debates, and revisions. The quality of identified evidence and the recommendations in the consensus are graded according to the Grading of Recommendations Assessment Development and Evaluation (GRADE system) [1, 2] (Table 1).

**Table 1:** Grades of evidence and strength of recommendation

Grade	Description
Quality of evidence	
High (A)	Further research cannot change the reliability of the therapeutic efficacy evaluation results
Medium (B)	Further research might change the reliability of the therapeutic efficacy evaluation results and also the evaluation results
Low or extremely low (C)	Further research will likely change the reliability of the therapeutic efficacy evaluation results and will likely also change the evaluation results
Strength of recommendation	
Strong (1)	Clearly indicates that the advantages of the intervention measures outweigh the disadvantages, or vice versa
Weak (2)	The advantages and disadvantages are indeterminate or the evidences, irrespective of the quality indicate that the advantages and disadvantages are equivalent

The Consensus aims to help physicians make reasonable decisions in the prevention, surveillance, and early diagnosis of HCC. However, it is neither mandatory, nor it is possible to include or solve all problems related to HCC. Therefore, clinicians should develop a comprehensive and reasonable chronic liver disease management and HCC monitoring regimen for specific patients based on the latest evidence-based medicine, their own expertise, clinical experience, and available medical resources. We will continue to improve the *consensus* based on relevant progress regionally and globally.

## Terms

Primary liver cancer: refers to primary malignant tumors in hepatocytes or intrahepatic biliary epithelial cells, mainly including HCC, ICC, and HCC–ICC. This *Consensus* mainly refers to HCC, the incidence of which is related to the hepatitis virus, cirrhosis, and dietary aflatoxins.

Risk factors: Factors that cause the occurrence of HCC or increase its probability, including viral infection, personal behaviors, lifestyle, environment, and genetics. Examples include HBV infection, HCV infection, alcohol intake, NAFLD/MAFLD, autoimmune liver disease, inherited metabolic liver disease, T2DM, and aflatoxin exposure.

Precancerous lesions: refer to benign lesions with cancerous potential. Precancerous lesions of HCC are defined as the formation of dysplastic nodules (DN) with a potential risk of malignant transformation due to atypia in tissue structure and cell morphology in the context of chronic liver disease, including low-grade dysplastic nodules (LGDN) and high-grade dysplastic nodules (HGDN), in order of increasing risk of malignant transformation.

Early HCC: HCC with a single cancerous nodule ≤ 5 cm in diameter or 2–3 cancerous nodules ≤ 3 cm in maximum diameter, without vascular invasion or extrahepatic metastasis, and can be treated radically. It includes stages 0–A on the Barcelona clinic liver cancer (BCLC) staging system or stages 1a–1b on the China liver cancer staging (CNLC) system.

Hepatobiliary-specific MRI contrast agents: these agents can be rapidly taken up by hepatocytes and transported to the extracellular space. By shortening the longitudinal relaxation time (T_1_) of tissue hydrogen, this enables hepatic angiography and cholangiography to be performed in a relatively short time window to detect and qualitatively diagnose focal liver lesions and evaluate liver function. New hepatobiliary-specific MRI contrast agents include gadolinium ethoxybenzyl diethylenetriamine pentaacetic acid (Gd-EOB-DTPA) and gadobenate dimeglumine (Gd-BOPTA).

Routine surveillance for HCC: According to the risk stratification of HCC in chronic liver disease, routine abdominal ultrasound and serum alpha-fetoprotein, or combined lens culinaris agglutinin-reactive fraction of AFP and des-gamma-carboxy prothrombin/protein induced by vitamin K absence or antagonist II, are used to screen and monitor the occurrence of HCC.

Enhanced surveillance for HCC: Based on the risk stratification of HCC in chronic liver disease and routine surveillance, liver CT and MRI plain scans and multi-phase dynamic contrast-enhanced imaging are used to screen and monitor the occurrence of HCC.

## Epidemiology

The WHO International Agency for Research on Cancer released the latest global cancer burden data in December 2020 (Globocan 2020) [3]. The incidence of primary liver cancer ranked sixth in malignant tumors, with 906,000 new cases, and mortality ranked third, with a total of 830,000 cases. The age-standardized incidence rate (ASIR) was 14.1/100,000 in men and 5.2/100,000 in women, with an overall mortality of 8.7/100,000. There were 657,000 new cases and 609,000 deaths in Asia, accounting for 72.5% and 73.3% worldwide, respectively. In particular, the incidence of primary liver cancer in China ranked fifth among malignant tumors, with 410,000 new cases, including 303,000 males. ASIR was 27.6/100,000 in males and 9.0/100,000 in females. Overall mortality ranked second, with 17.2/100,000 and 391,000 deaths. In the past 5 years, the average annual number of new cases of primary liver cancer was 995,000 cases worldwide, with 732,000 cases in Asia and 423,000 cases in China, accounting for 73.6% and 42.5% worldwide, respectively.

There are regional differences in the mean age of onset of HCC worldwide. The age of onset in Asian and African countries ranges from 30 to 60 years. According to the global HCC BRIDGE (“Bridge to Better Outcomes in HCC”) study [4], among the 18,031 HCC patients in 14 countries, the mean age of onset in Japan, Europe, and North America was 69, 65, and 62 years, respectively, and in China and South Korea, 52 and 59 years, respectively. In a Chinese study [5] that included 14,891 cases of HCC from 2016 to 2018, the proportion of patients ≤ 39, 40–49, 50–59, 60–69, and ≥ 70 years old was 2.89, 14.59, 29.47, 35.26, and 17.79%, respectively; the proportion of male and female patients was 76.01% and 23.99%, respectively.

The secondary prevention of HCC for the patients with chronic liver diseases focuses on surveillance, early diagnosis, and improving the cure rate and long-term survival. With the establishment of a nationwide liver cancer surveillance program in patients with hepatitis B and C in Japan and Taiwan, 60–73% and 70% of patients diagnosed with HCC were at the early stage (BCLC stages 0–A) in Japan and Taiwan, respectively, and 13% and 18% were in the advanced stage (BCLC stages C–D), respectively. In mainland China, the proportion of HCC patients diagnosed at BCLC stage 0, A, B, C, and D was 3, 30, 9, 55, and 2%, respectively, with an overall survival of 23 months and a 5-year survival of 11.7–14.1% [4, 6]. Therefore, it is urgent to implement standardized secondary prevention measures in the world.

## Causes and risk factors of HCC

Liver cirrhosis is a major risk factor for HCC. Chronic HBV infection is the main cause of HCC in China, accounting for approximately 86%. Other causes include chronic HCV infection, alcohol-related liver disease (ALD) caused by long-term excessive drinking, NAFLD, T2DM, and long-term consumption of aflatoxin-contaminated food.

### Liver cirrhosis

Approximately 7 million people (0.51%) in China suffer from liver cirrhosis, with an annual incidence of HCC ranging from 1 to 8% [7]. The 5-year cumulative incidence of HCC caused by cirrhosis with different etiologies is 30% for HCV infection, 15–17% for HBV infection, 8% for alcoholic cirrhosis, and 4% for primary biliary cirrhosis [8]. The annual incidence of HCC in patients with HBV-related cirrhosis who receive antiviral treatment is 1.5–2.5% [9]. The 7-year cumulative incidence of HCC in patients with cirrhosis associated with non-alcoholic steatohepatitis (NASH) is 2.4% [10]. In a cohort of 2079 patients with multi-etiology cirrhosis that was followed for 10 years, the 10-year cumulative incidence of HCC was 16.3% and 4.6% in patients with NASH-associated cirrhosis and autoimmune hepatitis induced cirrhosis, respectively [11].

### Chronic HBV infection

According to the World Health Organization, there are approximately 257 million chronic HBV patients worldwide. At present, the positive rate of serum HBsAg in the general population in China is 5–6%, and there are approximately 70 million patients with chronic HBV infection, including 20–30 million patients with chronic hepatitis B (CHB). Age > 40 years, male gender, Asian race, and family history of HCC are high-risk factors for the occurrence of HCC. Compared with the population under 40 years of age, risk increases 3.6, 5.1, and 8.3 times in the population aged 40–49, 50–59, and ≥ 60 years, respectively. The male-to-female risk ratio is about 3:1. The risk increases 5.6 times in the population with more than two HCC-diagnosed family members [12–14]. The risk of HCC in HBV-infected patients is 10–65 times higher than in non-HBV-infected patients in different regions of Asia [15]. In East Asia, the 5-year cumulative incidence of HCC in adult patients with HBV immune tolerance, CHB, and hepatitis B cirrhosis is 1, 3, and 17%, respectively [16].

### Chronic HCV infection

HCV infection is a worldwide epidemic, as there are 71 million people in the world with chronic HCV infection, including approximately 10 million people (0.72%) in China [17]. The risk of HCC in patients with chronic HCV infection is 5–20 times higher than in non-infected patients [11, 18]. The incidence of HCC increases with the progression of HCV-related liver fibrosis. The annual incidence of HCC in patients with fibrosis stages 1, 2, 3, and 4 is 0.5, 2.0, 5.3, and 7.9%, respectively [19]. The risk of HCC in patients infected with genotype 1b HCV is 1.78 times higher than in patients infected with HCV of other genotypes [20].

### ALD

The median prevalence of ALD in China is 4.5% (2.3–6.1%), with approximately 62 million cases [21]. Abstinence from alcohol reduces the risk of HCC by 6–7% per year, and a wash-out period of approximately 23 years is required to achieve the same risk as in non-drinkers [22]. The risk of HCC in drinkers with an alcohol consumption of 25, 50, and 100 g per day increases 1.19, 1.40, and 1.81 times, respectively, compared with non-drinkers [23]. Patients with HBV or HCV infection consuming more than 80 g of ethanol per day have a 53.9-fold increased risk of HCC; diabetic patients consuming more than 80 g of ethanol g/per day have a 9.9-fold increased risk of HCC [24].

### NAFLD, metabolic syndrome, and obesity

NAFLD has become the most prevalent chronic liver disease worldwide. Given its close correlation with overweight or obesity and glucose and lipid metabolism disorders, the international expert panel recommends renaming it as metabolic-associated fatty liver disease (MAFLD), and the Asian-Pacific Association for the Study of the Liver has developed relevant clinical practice guidelines. The prevalence in the general population in China is 15% (6.3–27.0%), approximately 173–338 million cases [25]. The incidence of HCC associated with NAFLD is 0.44 per 1000 person-years [26]. Chronic hepatitis C (CHC) patients with concomitant obesity (body mass index ≥ 30 kg/m^2^) have a 4.13-fold increased risk of HCC compared with non-obese patients, and those with concomitant T2DM have a 3.52-fold increased risk. In addition, CHB patients with T2DM have a 2.27-fold increased risk of HCC compared with non-diabetic patients, and HBV/HCV co-infected patients with obesity and T2DM have a more than a 100-fold increased risk [27].

### Carcinogen exposure

Aflatoxin B1, a metabolite produced by *Aspergillus flavus* and *Aspergillus parasiticus*, is highly carcinogenic. Areas with high aflatoxin exposure are also generally HBV-endemic, with a 73-fold increased risk of HCC in people exposed to both HBV and aflatoxin [28].

### Precancerous lesions

Hepatic precancerous lesions, often developing from chronic liver disease, are more common in patients with cirrhosis. Japanese scholars reported that the 1-, 3-, and 5-year cumulative incidences of HCC were 3.3, 9.7 and 12.4% in the patients with cirrhotic hyperplastic nodules, 2.6, 30.2, and 36.6% in LGDN patients and 46.2, 61.5, and 80.8% in the patients with HGDN (known as precancerous lesions). The annual incidence of HCC was about 10% and 20% in LGDN and HGDN patients, respectively [29].

- Recommendation 1: Hepatic cirrhosis from any cause carries the risk of HCC, while hepatitis B cirrhosis is the main cause of HCC, which requires intensive surveillance (A, 1).
- Recommendation 2: A combination of multiple etiologies or risk factors (such as chronic HBV or HCV infection concomitant with ALD, NAFLD, T2DM, or metabolic syndrome) can significantly increase the risk of HCC, and close monitoring is required for such populations (B, 1).
- Recommendation 3: Patients with radiologically confirmed precancerous lesions (LGDN and HGDN, LI-RADS [Liver Image Reporting and Data Management System] Level 4) are at extremely high risk of HCC, for whom nodule growth and pathological changes should be closely monitored (B, 1).

## Populations at risk for HCC

Persistent liver inflammation, repair, and fibrous hyperplasia as well as abnormal proliferation of hepatocytes induce the onset and development of HCC. The high-risk HCC is inconsistently defined in the Europe and US guidelines for the diagnosis and treatment of liver cancer. The *Consensus* stratifies the risk populations according to the risk of HCC, combined with the causes of HCC, epidemiological characteristics, and evidence-based medicine in China, for which the corresponding monitoring schemes are established [30].

1. Low-risk population: Patients aged < 30 years, at an early and stable stage of chronic liver disease, without obvious liver inflammation and fibrosis, including chronic inactive HBsAg carriers, patients in the hepatitis B immune control period and with fatty liver disease. Also includes Gilbert syndrome, Dubin–Johnson syndrome, benign recurrent intrahepatic cholestasis, and other benign inherited metabolic liver diseases.
2. Moderate-risk population: CHB patients aged > 30 years (no family history of liver cancer, no long-term alcohol abuse, no smoking, no history of exposure to carcinogenic poisons, no concomitant diabetes or obesity), and other patients with active chronic liver disease including CHC, ALD, NASH, autoimmune liver disease, or Wilson's disease.
3. High-risk population: Patients meeting any of the following items: ① cirrhosis due to various causes including HBV infection, HCV infection, ALD, NAFLD, drug-induced liver injury, autoimmune liver disease, and Wilson's disease; ② CHB patients aged ≥ 30 years with a family history of liver cancer, or long-term alcohol abuse, smoking, clear history of exposure to carcinogenic poisons, concomitant T2DM, or obesity.
4. Extremely high-risk population: The high-risk population has one or more of the following items: ① imaging examinations such as ultrasound indicate suspected precancerous lesions or atypical space-occupying lesions in the liver; ② serum AFP ≥ 20 ng/ml, with or without DCP ≥ 40 mAU/ml and/or AFP-L3 ≥ 15%; ③ dysplastic nodules of the liver confirmed by imaging or liver histopathology.

## Secondary prevention measures for primary liver cancer

### Surveillance tests

The occurrence and stage of HCC are comprehensively evaluated and monitored using serum marker levels and imaging findings. If necessary, liver biopsy is performed to determine the nature, differentiation, and gene expression of the nodules.

#### Serum markers

##### AFP

AFP was first detected in the serum of liver cancer in 1964 and has been routinely applied in HCC surveillance. With the cut-off value of AFP at 5 ng/ml, the sensitivity and specificity are 62% and 87% for early HCC (< 2 cm), while the cut-off value is 20 ng/ml, and the sensitivity and specificity are 52.9% and 93.3% for HCC [31, 32]. However, with the rapid development of medical imaging, the proportion of diagnosed small hepatocellular carcinoma is increasing, and the sensitivity of AFP is gradually decreasing, with AFP at normal or low levels in 30–40% of HCC patients. Furthermore, antiviral treatment in CHB and CHC patients indeed reduces the AFP levels, persistent AFP elevation after antiviral 12 months is significantly associated with the HCC development. CHB patients with persistent AFP positivity would have 6.35-fold (8.9% vs 1.4%) higher risk for developing HCC compared with CHB patients with normalized AFP. This risk was even higher in cirrhotic CHB patients with non-AFP response (43.48%, 10/23) comparing AFP normalized cirrhotic patients (0, 0/18), *p* < 0.005 [33]. It is similar for the chronic hepatitis C patients with DAAs, and persistent (> 12 months) AFP elevation might serve as an indicator of HCC development [34]. HCC high-risk patients with normal or mildly elevated serum AFP should be comprehensive assessed by dynamic observation combined with virological response, liver biochemistry, imaging, and liver biopsy findings.

##### DCP/PIVKA-II

DCP/PIVKA-II is a liver-synthesized abnormal prothrombin that lacks coagulation activity. In 1984, Liebman et al. first found serum DCP significantly increased in HCC patients and proposed it as a novel tumor marker for HCC. A recent study reported that the sensitivity and specificity of PIVKA-II ≥ 40 mAU/ml in the diagnosis of early HCC were 64% and 89%, respectively, with an accuracy of 86.3% [31].

##### AFP-L3

AFP-L3 is a glycoprotein mainly derived from hepatoma cells that is not affected by AFP level. In 2005, FDA approved AFP-L3 as a HCC surveillance indicator, and AFP-L3 ≥ 10% as the cut-off value for the diagnosis of HCC. Studies reported that 34.3% of HCC patients with normal AFP developed an AFP-L3 abnormality as early as 1 year before diagnosis [35]. Meta-analysis showed that the sensitivity and specificity of AFP-L3 in the diagnosis of HCC were 48.3% (45.9–50.7%) and 92.9% (91.6–94.0%), respectively. Combined detection of AFP and AFP-L3 can improve the diagnostic rate of early HCC [36].

##### Other markers

Combined detection of carbohydrate antigen 199 with AFP and carcinoembryonic antigen can improve the diagnostic rate of HCC. In addition, there are a variety of novel serological molecular markers under exploration such as glypican-3, Golgi protein 73, α-L-fucosidase, osteopontin, microRNA, circulating tumor cells, circulating tumor DNA, exosomes, and circulating tumor DNA methylation. These all have specific advantages in the diagnosis of HCC, but they also have limitations. Detecting one of the above markers is not sufficient for ideal diagnostic efficiency. Combined detection of different markers may be an effective measure to improve the detection rate of early HCC, which should be proved by further study.

- Recommendation 4: AFP remains the preferred serological marker for early HCC surveillance (A, 1), and combined detection with PIVKA-II and AFP-L3 can improve the diagnostic accuracy (B, 2).
- Recommendation 5: For patients with negative or mildly elevated serum AFP, combined detection of PIVKA-II and AFP-L3, based on the dynamic change of serum AFP levels, can improve the diagnostic accuracy of early HCC (B, 2).

#### Imaging examination

**Fig. 2:**
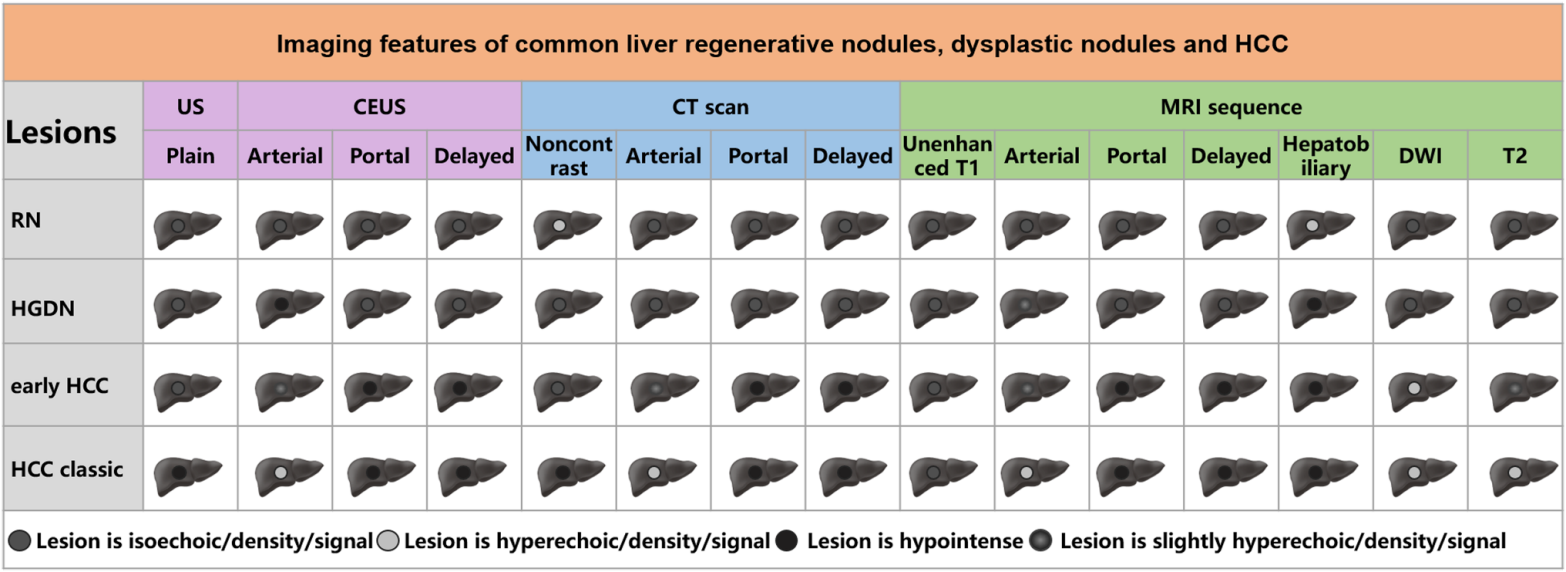
Schematic diagram of imaging features of liver regenerative nodules, dysplastic nodules, and HCC. *ASH* alcoholic hepatitis; *AIH* autoimmune hepatitis; *PBC* primary biliary cholangitis

Imaging examination is an important means of HCC surveillance. Routine ultrasound, contrast-enhanced ultrasonography (CEUS), CT, MRI, digital subtraction angiography (DSA), positron emission tomography (PET), and PET/CT are commonly used methods, which have distinct advantages and disadvantages for assessing focal liver lesions (Fig. 2). CT and MRI are often used for confirmative diagnosis of abnormity suggested by the initial ultrasound test.

##### Routine abdominal ultrasound

Abdominal ultrasound is easy to perform, convenient to operate, non-invasive, and repeatable. As the most used HCC screening and detection method, it has a high sensitivity for liver space-occupying lesions > 2 cm in diameter, and its sensitivity increases with tumor volume. The overall sensitivity of ultrasound in the diagnosis of HCC at various clinical stages is approximately 84%, but the sensitivity for early HCC is 32–63%, with a specificity of 91–95%, and the sensitivity can be increased to 70% in combination with AFP [37, 38].

##### Liver CEUS

CEUS typically presents as “fast in and fast out.” The arterial phase shows homogeneous or inhomogeneous enhancement, and the portal phase and delayed phase are hypoechoic or even anechoic. It is reported that the sensitivity, specificity, and accuracy of CEUS in the diagnosis of HCC were 95.3, 100, and 98.1%, respectively [39]. In addition, the sensitivity and specificity for early HCC were 80–94% and 82–98%, respectively, and for small hepatocellular carcinoma (diameter ≤ 2 cm), they were 63–70% and 89–93%, respectively [40, 41].

##### Liver CT

CT can detect solid tumors > 1 cm in diameter. Typical signs of HCC on CT include: non-contrast scans showing lower density compared with the normal liver; obvious enhancement of arterial phase with high density which decreases rapidly with the outflow of contrast agent; and tumor enhancement in the portal venous phase and/or equilibrium phase lower than in the liver parenchyma (showing the characteristics of “fast in and fast out”). The sensitivity and specificity of enhanced CT in the diagnosis of HCC are 66–79% and 90–94%, respectively [42, 43].

##### Liver MRI

MRI has high contrast resolution for soft tissues and is characterized by multi-parameter, multi-directional, and multi-sequence imaging. MRI is more sensitive and accurate than CT for the diagnosis and differential diagnosis of liver nodules. Conventional extracellular fluid contrast-enhanced MRI has a sensitivity of 84–90% and a specificity of 83–94% in the diagnosis of early HCC [42–44]. Typical manifestations of HCC on MRI include: high signal intensity on T_1_ and T_2_; dynamic enhanced scan showing significant enhancement or inhomogeneous enhancement in the arterial phase, rapid wash-out in the portal venous phase and/or delayed phase with isointensity or hypointensity; and some patients show tumor peripheral pseudocapsule images. Diffusion-weighted imaging (DWI) sequence shows hyperintense lesions. Hepatocyte-specific contrast-enhanced MRI can improve the diagnostic rate of early HCC, as the uptake rate of Gd-EOB-DTPA and Gd-BOPTA in hepatocytes is 50% and 5%, respectively. Another manifestation of HCC may be that the hepatobiliary-specific phase shows a hypointense mass due to the lack of uptake of the contrast medium by the tumor, with a development time of 20–40 min and 40–120 min for Gd-EOB-DTPA and Gd-BOPTA, respectively. Gd-EOB-DTPA is widely used.

The sensitivity and specificity of Gd-EOB-DTPA-enhanced MRI in the diagnosis of small hepatocellular carcinoma (≤ 2 cm) are 90–96% and 87–96.6%, respectively [42, 45]. Additionally, hepatocellular carcinoma ≤ 1.0 cm in diameter can also be identified. Cirrhotic nodules, LGDN, HGDN, and early HCC can also be identified in combination with DWI. Gd-EOB-DTPA-enhanced MRI can aid in qualitative diagnosis when liver CT reveals a lesion rich in blood supply in the arterial phase with insignificant clearance in the portal and delayed phases and atypical HCC.

##### PET and PET/CT

PET/CT simultaneously obtains PET functional metabolic images and CT anatomical images to more accurately locate and characterize lesions with complementary advantages. However, due to the active metabolism of the liver itself, PET/CT has a sensitivity of only 55% in the diagnosis of liver cancer. It is not recommended for the screening and diagnosis of early HCC, but can be used to assess lymph-node metastasis and distant organ metastasis.

##### DSA

DSA is a significant tool for the diagnosis and differential diagnosis of small intrahepatic tumors. It can visualize the staining of liver tumors and blood vessels, and identify the number, size, and blood supply of tumors, thereby providing accurate and objective information on the anatomical relationship between liver tumors and important blood vessels, vascular anatomical variations, and portal vein invasion.

#### Liver biopsy

Liver biopsy is suitable for intrahepatic nodules lacking typical imaging features. Biopsy can clarify the nature of the lesion, differentiate benign proliferative lesions such as focal proliferative nodules from early HCC, and can identify their molecular phenotypes to guide treatment and prognosis.

### Assessment of liver stiffness and reserve function

The evaluation of HCC risk factors is an important measure for early detection and prediction of the disease. The degree of disease progression, liver reserve function, the presence or absence of other diseases, and immune status are all related to the occurrence of HCC. Complete blood counts, liver biochemical indicators, blood lipid and blood glucose levels, coagulation function, and the progression of liver fibrosis should be monitored every 3–6 months. Liver functional reserve reflects the degree of liver injury. The sensitivity of ultrasound in the diagnosis of early liver cancer is 60% in patients with MELD ≥ 10 and only 18.8% in those with lower scores [46]. Liver stiffness measurement (LSM) detected by transient elastography and FIB-4 index are helpful for predicting the risk of HCC. The Korean study reported that the risk of HCC increased by 3.07, 4.68, 5.55, and 6.60 times in patients with chronic HBV infection when LSM was 8.1–13, 13.1–18, 18.1–23, and > 23 kPa, respectively [47]. Among HCV-infected patients in Taiwan, the 5-year cumulative incidence of HCC was 0.9, 9.5, and 45.1% in patients with LSM < 12.0, 12.0–24.0, and > 24.0 kPa, respectively [48]. NAFLD patients with FIB-4 of 1.30–2.67 (moderate liver fibrosis) and > 2.67 (severe liver fibrosis/cirrhosis) had a 3.74- and 25.2-fold increased risk of HCC compared with those with FIB-4 < 1.3 (no significant fibrosis) [49]. Therefore, accurate evaluation of the degree of liver fibrosis has a certain clinical value for predicting the risk of HCC.

- Recommendation 6: Routine abdominal ultrasound is the main imaging method for monitoring the HCC risk population; it can detect tumors and nodules > 2 cm. In addition, CEUS can assist in differentiating tumor features (A, 1).
- Recommendation 7: Plain and enhanced liver CT, an important imaging method for the early surveillance of HCC, can be used for the differential diagnosis and monitoring of nodules > 1 cm in diameter (A, 1).
- Recommendation 8: Multimodal MRI (plain, DWI, and enhanced) is the most sensitive imaging method for the surveillance of HCC. It can detect tumors ≤ 1 cm in diameter and is used for HCC surveillance in nodular cirrhosis and to differentiate the features of suspicious nodules found by ultrasound. Hepatocyte-specific Gd-EOB-DTPA-enhanced MRI can improve the detection rate of HCC with the diameter ≤ 1 cm and is a valuable clinical means to differentiate benign hyperplastic nodules, precancerous lesions, and early HCC (A, 1).
- Recommendation 9: Accurate assessment of liver functional reserve and liver stiffness can be referred for predicting the risk of HCC development due to chronic liver disease (C, 2).

### Liver cancer surveillance

The risk of HCC gradually increases with the age of patients with chronic liver disease. According to the annual report of global cancer, mainland China, with an average annual increase of more than 2%, was among the regions with the largest increase between 2007 and 2017. Standardized surveillance is the key approach to early detection, diagnosis, and radical cure of HCC. Recently, a total of 18,816 CHB patients were screened with the above methods every 6 months and followed up for 5 years. The 1-, 2-, and 5-year survival of the 87 HCC patients were 65.9, 52.6, and 46.4% in the screening group, and 31.2, 7.2, and 0% in the control group, respectively. Mortality related to liver cancer was 83.2/100,000 in the screening group and 131.5/100,000 in the control group, and the risk of death was 0.63:1 [50]. A total of 173,378 high-risk patients with chronic liver disease (cirrhosis) in Japan were screened by ultrasound combined with AFP, AFP-L3, and PIVKA-II every 3–4 months, and the extremely high-risk population was screened by Gd-EOB-DTPA-enhanced MRI or multi-slice spiral CT every 6–12 months. The diagnostic rate of early HCC was 62%, and the 5-year survival was 42.7% [51].

**Fig. 3:**
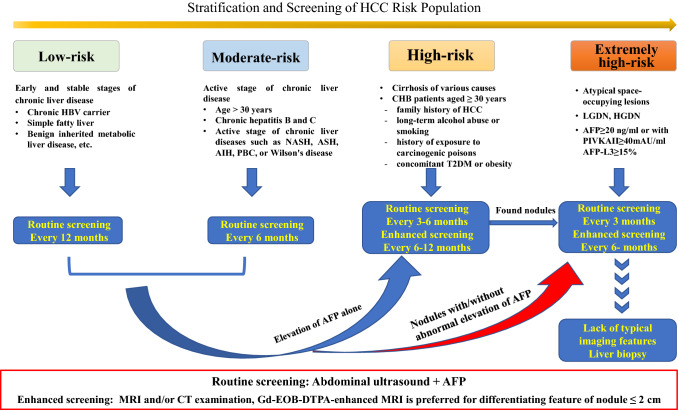
Flowchart of stratification and screening of HCC in at-risk populations. *ASH* alcoholic hepatitis; *AIH* autoimmune hepatitis; *PBC* primary biliary cholangitis

According to the risk stratification of HCC in patients with chronic liver disease, it is recommended to use abdominal ultrasound and serum AFP for routine surveillance, and multimodal MRI or CT enhanced surveillance are recommended for the high-risk population. (Fig. 3).

- Recommendation 10: Abdominal ultrasound combined with AFP is a routine surveillance method for HCC in patients with chronic liver disease, and multimodal liver MRI and/or CT are enhanced surveillance methods. Routine surveillance is performed once a year for the low-risk population and every 6 months for the moderate-risk population (C, 1); routine surveillance every 3–6 months (A, 1) and enhanced surveillance every 6–12 months (B, 2) are performed for the high-risk population; routine surveillance every 3 months and enhanced surveillance every 6 months are performed for the extremely high-risk population (B, 1).
- Recommendation 11: If an abdominal ultrasound shows nodules < 1 cm during monitoring, reexamination should be performed every 3 months. If the nodule grows or the nodule is > 1 cm and AFP > 20 ng/ml, the enhanced surveillance process for HCC should be initiated. If the nature of the nodule cannot be determined by imaging examination, image-guided diagnostic liver biopsy may be considered (C, 1).

### Treatment and intervention for HCC-related diseases

#### Etiological treatment

##### Antiviral therapy for chronic HBV and HCV infection

Nucleos(t)ide analogues (NAs) and pegylated interferon-α (PEG-IFNα) are two major types of antiviral drugs for chronic HBV infection. After treatment with PEG-IFNα (relative risk ratio = 0.66), risk of HCC was reduced in patients with chronic HBV infection, which was more significant in patients with liver cirrhosis (relative risk ratio = 0.53) [52]. The results of the Taiwan clinical cohort study suggested that NAs could reduce the risk of HCC in patients with chronic HBV infection (risk ratio = 0.37) [53]. The patients with CHB, ALT ≥ 1 × ULN or cirrhosis should be considered for antiviral therapies according to the protocols outlined in Asian-Pacific clinical practice guidelines on the management of hepatitis B (a 2015 update) [54] or the National Guidelines for the prevention and treatment of chronic hepatitis B.

Antiviral therapy for CHC has entered the era of pan-genotypic direct-acting antiviral drugs (DAA) such as sofosbuvir/velpatasvir and glecaprevir/pibrentasvir. DAA treatment can reduce the risk of HCC in patients with chronic HCV infection (adjusted risk ratio = 0.66) [55]. All chronic HCV patients with detectable HCV RNA should receive DAAs according to the APASL Consensus Statements and Recommendation on Treatment of Hepatitis C [56] or the National Guidelines for the prevention and treatment of hepatitis C. Patients with hepatitis C cirrhosis achieved sustained virological response (SVR) after DAA treatment, and the incidence of HCC was still significantly higher than that of non-cirrhotic patients who achieved SVR (risk ratio = 4.73) [57]. Shiha et al. reported that, in CHC patients with HCV-infected advanced liver fibrosis, the incidence of HCC was 2.917/100 PY in patients with cirrhosis, while in patients with advanced liver fibrosis, the incidence of HCC was 0.664/100 PY [58]. Therefore, patients with advanced fibrosis and hepatitis C cirrhosis should monitor the HCC occurrence after achieving SVR with DAA treatment.

##### ALD

Abstinence from alcohol is the most important and basic treatment for ALD, which can reduce liver histological damage, delay the process of fibrosis, and improve the survival of ALD patients. HCC risk was reduced 6–7% every year in ALD patients after abstinence from alcohol [22].

##### NAFLD

To date, there are no effective drugs recommended for the prevention of HCC in NAFLD patients. The risk of HCC can be reduced by controlling body weight, reducing waist circumference, correcting lipid metabolic disorders, and reducing liver inflammation and fibrosis through measures such as changing unhealthy lifestyles and increasing aerobic exercise.

##### Diabetes mellitus

For patients with chronic liver disease and T2DM, individualized lifestyle intervention and hypoglycemic drugs are combined to strictly control blood glucose levels. Metformin has been shown to significantly reduce the risk of HCC in patients with chronic liver disease and diabetes mellitus.

##### Improve living environment

Reducing exposure to aflatoxins can significantly reduce the population-wide morbidity and mortality of HCC. In people exposed to aflatoxin, oltipraz or chlorophyllin is protective [59, 60].

#### Anti-inflammatory and anti-fibrotic therapy

Anti-inflammatory and liver-protecting drugs selected based on the characteristic of liver inflammation due to different etiologies and drug function can reduce disease progression.

- Recommendation 12: The risk of HCC can be reduced when sustained virological response is achieved via antiviral therapies with NAs or PEG-IFNα in patients with CHB, or DAAs in patients with CHC. However, the risk cannot be completely eliminated by antiviral therapies, especially for patients with liver cirrhosis. Therefore, it is necessary to monitor the occurrence of HCC according to the surveillance programs after achieving virological response (A, 1).
- Recommendation 13: Abstinence from alcohol can reduce the risk of HCC in patients with ALD (A, 1).
- Recommendation 14: In patients with NAFLD, the risk of HCC can be reduced by changing unhealthy lifestyles, increasing aerobic exercise, and other measures for controlling body weight and preventing and treating metabolic disorders (B, 1).
- Recommendation 15: Patients with both chronic liver disease and T2DM face increased risk of HCC, and therefore, blood glucose levels should be strictly monitored and controlled (B1).

## Clinical issues to be investigated and addressed

1. Serological markers with high sensitivity and specificity for the surveillance of early HCC.
2. Multicenter clinical studies focused on prevention of HCC via treatment of chronic viral hepatitis using antiviral therapies and anti-fibrosis treatment.
3. Studies on reducing the risk of HCC associated with hepatitis B and C through combination therapy with antiviral and immunomodulatory drugs.
4. The cumulative incidence of HCC in CHB, CHC with NAFLD, and ALD.
5. Elimination of the effects of metabolism-related fatty liver disease on the incidence of HCC.
6. Health economic evaluation of the implementation of liver cancer screening programs.

## Abbreviations

HCC: Hepatocellular carcinoma
ICC: Intrahepatic cholangiocarcinoma
NAFLD: Non-alcoholic fatty liver disease
MAFLD: Metabolic-associated fatty liver disease
T2DM: Type 2 diabetes mellitus
DN: Dysplastic nodules
LGDN: Low-grade dysplastic nodules
HGDN: High-grade dysplastic nodules
BCLC: Barcelona clinic liver cancer staging
CNLC: China liver cancer staging
Gd-EOB-DTPA: Gadolinium ethoxybenzyl diethylenetriamine pentaacetic acid
AFP: Alpha-fetoprotein
DCP: Des-gamma-carboxy prothrombin
PIVKA-II: Protein induced by vitamin K absence or antagonist II
AFP-L3: Lens culinaris agglutinin-reactive fraction of AFP
NASH: Non-alcoholic steatohepatitis
CEUS: Contrast-enhanced ultrasonography
LSM: Liver stiffness measurement
NAs: Nucleoside (acid) analogues
DAA: Direct-acting antiviral drugs
SVR: Sustained virological response
