# Correction to: Consensus on the secondary prevention of primary liver cancer

**DOI:** 10.1007/s12072-021-10288-2

**Published:** 2022-01-12

**Authors:** Yuemin Nan, Xiaoyuan Xu, Yanhang Gao, Rongqi Wang, Wengang Li, Ming Yang, Lingdi Liu, Zhongping Duan, Jidong Jia, Lai Wei, Hui Zhuang, Chinese Society of Hepatology, Chinese Medical Association, Huiguo Ding, Zhongping Duan, Jiangao Fan, Qinmao Fang, Yanhang Gao, Peng Hu, Jidong Jia, Wengang Li, Jingfeng Liu, Junqi Niu, Yuemin Nan, Jia Shang, Rongqi Wang, Lai Wei, Yanyan Yu, Yuguo Zhang, Suxian Zhao, Jian Zhou, Weifeng Zhao, Xiaoyuan Xu, Chuanmiao Xie, Wen Xie, Ming Yang, Hui Zhuang

**Affiliations:** 1 Present Address: Department of Traditional and Western Medical Hepatology, The Third Hospital of Hebei Medical University, Shijiazhuang, 050051 China; 2 Department of Infectious Diseases, Peking University First Hospital, Beijing, 100034 China; 3 Department of Hepatology, The First Hospital of Jilin University, Changchun, 130021 China; 4 Radiation Oncology Centre, The Fifth Medical Centre of Chinese PLA General Hospital, Beijing, 100039 China; 5 Peking University Hepatology Institute, Peking University People’s Hospital, Beijing, China; 6 Artificial Liver Centre, Beijing You-An Hospital, Capital Medical University, Beijing, China; 7 Liver Research Centre, Beijing Friendship Hospital, Capital Medical University, Beijing, China; 8 Hepatopancreatobiliary Centre, Beijing Tsinghua Changgung Hospital, Tsinghua University, Beijing, China; 9 Department of Microbiology and Centre for Infectious Diseases, Peking University Health Science Centre, Beijing, China

## Correction to: Hepatology International (2021) 15:1289–1300 10.1007/s12072-021-10259-7

The article [Consensus on the secondary prevention of primary liver cancer], written by [Yuemin Nan, Xiaoyuan Xu, Yanhang Gao, Rongqi Wang, Wengang Li, Ming Yang, Lingdi Liu,Zhongping Duan, Jidong Jia, Lai Wei, Hui Zhuang, Chinese Society of Hepatology, Chinese Medical Association], was originally published Online First without Open Access. After publication in volume [15], issue [6], page [1289–1300] the author decided to opt for Open Choice and to make the article an Open Access publication. Therefore, the copyright of the article has been changed to © [The Author(s)] [2021] and the article is forthwith distributed under the terms of the Creative Commons Attribution [This article is licensed under a Creative Commons Attribution 4.0 International License, which permits use, sharing, adaptation, distribution and reproduction in any medium or format, as long as you give appropriate credit to the original author(s) and the source, provide a link to the Creative Commons licence, and indicate if changes were made. The images or other third party material in this article are included in the article’s Creative Commons licence, unless indicated otherwise in a credit line to the material. If material is not included in the article’s Creative Commons licence and your intended use is not permitted by statutory regulation or exceeds the permitted use, you will need to obtain permission directly from the copyright holder. To view a copy of this licence, visit http://creativecommons.org/licenses/by/4.0/.

